# Impact of DNA Demethylases on the DNA Methylation and Transcription of *Arabidopsis NLR* Genes

**DOI:** 10.3389/fgene.2020.00460

**Published:** 2020-05-26

**Authors:** Weiwen Kong, Xue Xia, Qianqian Wang, Li-Wei Liu, Shengwei Zhang, Li Ding, Aixin Liu, Honggui La

**Affiliations:** ^1^School of Horticulture and Plant Protection, Yangzhou University, Yangzhou, China; ^2^Joint International Research Laboratory of Agriculture and Agri-Product Safety of the Ministry of Education, Yangzhou University, Yangzhou, China; ^3^College of Life Sciences, Nanjing Agricultural University, Nanjing, China; ^4^State Key Laboratory of Pharmaceutical Biotechnology, School of Life Sciences, Nanjing University, Nanjing, China; ^5^Department of Plant Protection, Shandong Agricultural University, Tai’an, China

**Keywords:** nucleotide-binding leucine-rich repeat genes, DNA demethylases, cytosine methylation, active DNA demethylation, transcriptional regulation

## Abstract

Active DNA demethylation is an important epigenetic process that plays a key role in maintaining normal gene expression. In plants, active DNA demethylation is mediated by DNA demethylases, including ROS1, DME, DML2, and DML3. In this study, the available bisulfite sequencing and mRNA sequencing data from *ros1* and *rdd* mutants were analyzed to reveal how the active DNA demethylation process shapes the DNA methylation patterns of *Arabidopsis* nucleotide-binding leucine-rich repeat (*NLR*) genes, a class of important plant disease resistance genes. We demonstrate that the CG methylation levels of three *NLR* genes (*AT5G49140*, *AT5G35450*, and *AT5G36930*) are increased in the *ros1* mutants relative to the wild-type plants, whereas the CG methylation level of *AT2G17050* is decreased. We also observed increased CG methylation levels of *AT4G11170* and *AT5G47260* and decreased CG methylation levels of *AT5G38350* in *rdd* mutants. We further found that the expression of three *NLR* genes (*AT1G12280*, *AT1G61180*, and *AT4G19520*) was activated in both *ros1* and *rdd* mutants, whereas the expression of another three *NLR* genes (*AT1G58602, AT1G59620*, and *AT1G62630*) was repressed in these two mutants. Quantitative reverse transcriptase–polymerase chain reaction detection showed that the expression levels of AT1G58602.1, AT4G19520.3, AT4G19520.4, and AT4G19520.5 were decreased in the *ros1* mutant; AT3G50950.1 and AT3G50950.2 in the *rdd* mutant were also decreased in expression compared to Col-0, whereas AT1G57630.1, AT1G58602.2, and AT5G45510.1 were upregulated in the *rdd* mutant relative to Col-0. These results indicate that some *NLR* genes are regulated by DNA demethylases. Our study demonstrates that each DNA demethylase (ROS1, DML2, and DML3) exerts a specific effect on the DNA methylation of the *NLR* genes, and active DNA demethylation is part of the regulation of DNA methylation and transcriptional activity of some *Arabidopsis NLR* genes.

## Introduction

Cytosine DNA methylation is an important epigenetic mark (Johnson et al., 2002). It is observed on three sequence contexts, that is, CG, CHG, and CHH (where H represents A, C, or T), in the *Arabidopsis* genome (Chan et al., 2005). The regulation of gene expression by DNA methylation in plants has been discovered to play important roles in the cellular response to pathogen attacks (Dowen et al., 2012; Yu et al., 2013; Le et al., 2014; Deleris et al., 2016). DNA methylation patterns in eukaryotes are shaped by DNA methylation and demethylation processes (Chan et al., 2005; Meyer, 2011; Zhang et al., 2018).

It has been demonstrated that a plant-specific pathway, RNA-directed DNA methylation (RdDM), mediates *de novo* cytosine methylation in three cytosine sequence contexts (Zhang and Zhu, 2011). More studies have revealed that two RdDM mechanisms, canonical and non-canonical RdDM pathways, establish DNA methylation in plants (Matzke and Mosher, 2014). In RdDM pathways, the *de novo* methyltransferase DRM1/2 plays key roles in sequence-specific cytosine methylation. Additionally, cytosine methylation has been determined to be established and maintained through several key methyltransferases in plants (Bender, 2004; Chan et al., 2005; Law and Jacobsen, 2010).

In *Arabidopsis* (*Arabidopsis thaliana*), active DNA demethylation is mediated by DNA glycosylase/lyases, that is, ROS1, DME, DML2, and DML3 (Choi et al., 2002; Penterman et al., 2007; Zhu, 2009). It is known that *Arabidopsis* ROS1 (repressor of silencing 1), a bifunctional DNA glycosylase/lyase, functions in repressing transcriptional gene silencing by the action of DNA demethylation (Gong et al., 2002). Mutations in ROS1 result in DNA hypermethylation and transcriptional silencing of specific genes (Penterman et al., 2007). It has been shown that hypermethylation is triggered in the promoters of some silenced loci in *ros1* mutants (Gong et al., 2002). *Arabidopsis DME* encodes a protein containing a DNA glycosylase domain and a nuclear localization domain, which is able to actively erase 5-methylcytosines by a base excision repair pathway (Choi et al., 2002; Morales-Ruiz et al., 2006). Another two DME paralogs, known as demeter-like proteins DML2 and DML3, were found in the genome of *Arabidopsis* (Choi et al., 2002; Penterman et al., 2007). DMEs function mainly in the central cells of female gametophytes, and they are vital for imprinted genes, for example, *MEA*, to be expressed in a maternal allele-specific pattern in the endosperm (Gehring et al., 2006; Bauer and Fischer, 2011). The other three demethylases, ROS1, DML2, and DML3, were shown to be largely active in *Arabidopsis* somatic cells (Gong et al., 2002; Ortega-Galisteo et al., 2008). It was found that approximately 180 discrete loci throughout the *Arabidopsis* genome were demethylated by DML enzymes, and more than 80% of these loci were located in genic regions (Penterman et al., 2007). Strikingly, the 5′ and 3′ ends of these regions were primarily targeted by the DML enzymes (Penterman et al., 2007). DML3 was also observed to demethylate preferentially symmetrical sequence contexts (CpG and CpHpG) (Ortega-Galisteo et al., 2008). *rdd* is a triple mutant with mutations in ROS1, DML2, and DML3 (Penterman et al., 2007). It was reported that many hypermethylated regions in *rdd* do not overlap with those in *ros1* (Qian et al., 2012). This finding suggests that DML2 and DML3 have specific functions in contrast to ROS1. An earlier study demonstrated that after DNA demethylation occurred in *Arabidopsis*, activation of the defense response mediated by salicylic acid was observed, and bacterial pathogen multiplication was restricted (Yu et al., 2013). Another study revealed that stress-responsive genes in *Arabidopsis* can be modulated by DNA demethylases by targeting transposable elements within their promoters (Le et al., 2014). These results imply that active DNA demethylation is a factor that strongly affects disease resistance in plants.

Nucleotide-binding leucine-rich repeat (NLR) proteins, a class of immune receptors, play an important part in plant disease resistance. It was reported that approximately 150 typical *Arabidopsis NLR* genes were identified and characterized in ecotype Col-0 (Meyers et al., 2003). All the proteins were categorized into Toll/interleukin 1 receptor (TIR) or coiled-coil (CC) motif-containing NLR subfamilies, abbreviated as TNL and CNL, respectively (Meyers et al., 2003; McHale et al., 2006). Plant *NLR* genes have been well-known to play fundamental roles in disease resistance (Dangl and Jones, 2001). However, the transcriptional regulation of *NLR* genes has not been thoroughly elucidated, despite their importance in plant disease resistance. The expression levels of plant *NLR* genes may be regulated by diverse factors, including tissue types, developmental stages, environmental cues, and pathogen attacks (Yoshimura et al., 1998; Wang et al., 1999). A previous study revealed that most *Arabidopsis NLR* genes were expressed weakly, even with tissue-specific expression patterns (Tan et al., 2007). Some evidence has shown that small RNAs modulate the expression of plant *NLR* genes (Zhai et al., 2011; Li et al., 2012; Shivaprasad et al., 2012; Fei et al., 2013). Phased, secondary, small interfering RNAs (phasiRNAs), formerly known as *trans-*acting small interfering RNAs (tasiRNAs), are primed by miRNAs, a category of small RNAs. phasiRNAs and miRNAs were found to suppress the expression of tomato *NLR* genes (Shivaprasad et al., 2012). It was reported that an *Arabidopsis NLR* gene, *At4g11170*, temporarily named *resistance methylated gene 1* (*RMG1*) by the authors, is an outstanding RdDM target, and ROS1 is essential for its background expression and activated transcription (Yu et al., 2013). *RBA1*, encoding a TIR-containing, truncated NLR protein, is speculated to be regulated through cytosine methylation in the *Arabidopsis* Col-0 ecotype (Nishimura et al., 2017). In addition, new findings suggested that DNA methylation is involved in regulating the expression of some *NLR* genes in *Arabidopsis* and common bean (Kong et al., 2018; Richard et al., 2018).

A previous study demonstrated that single, double, and triple F2 mutants of *ROS1*, *DML2*, and *DML3* show no obvious morphological phenotypes under their growth conditions (Penterman et al., 2007; Ortega-Galisteo et al., 2008). However, developmental abnormalities were observed in some *ros1* mutants in later generations (Gong et al., 2002). Furthermore, the *ros1* mutant is sensitive to hydrogen peroxide and methyl methanesulfonate (Gong et al., 2002). Additionally, it was observed that a slightly increased bacterial growth occurred in the *ros1* mutant, but not in the *dml2* and *dml3* mutants with inoculation of *Pseudomonas syringae* pv. *tomato* strain DC3000 (Yu et al., 2013). In the *rdd* mutant, an enhanced susceptibility was found to *Fusarium oxysporum* (Le et al., 2014). Another study showed that opposite phenotypes were observed in *Arabidopsis* hypomethylated mutants and hypermethylated mutants after infection with *Hyaloperonospora arabidopsidis* (Lopez Sanchez et al., 2016).

In this study, we used publicly available bisulfite sequencing (BS-Seq) data to identify *Arabidopsis NLR* genes that are targeted by demethylases, including ROS1, DML2, and DML3 in wild-type plants. We demonstrate that the CG methylation levels in the 5′ upstream regions (UPRs) of 30 *Arabidopsis NLR* genes were increased in both the *ros1* and *rdd* mutant plants. Furthermore, we show that 32 *Arabidopsis NLR* genes were presumably regulated by both ROS1 and DML demethylases at the transcriptional level. In conclusion, our data indicate that active DNA demethylation by ROS1 and DML enzymes functions to protect *Arabidopsis NLR* genes from potentially deleterious methylation. The data also implicate ROS1 and DML demethylases in determining the DNA methylation profiles of *Arabidopsis NLR* genes. Additionally, we analyzed the available mRNA-Seq data from *Arabidopsis ros1*, *rdd* mutants, and their wild-type control plants. We found that mutations in DNA demethylases lead to changes in the transcriptional activities of some *Arabidopsis NLR* genes, suggesting that their expression is regulated by DNA demethylases.

## Materials and Methods

### Retrieval of *Arabidopsis* BS-Seq and mRNA-Seq Data

The *Arabidopsis* BS-Seq data used in this study were retrieved from the Gene Expression Omnibus (GEO) database.^1^ The GEO accession numbers for the data are GSM1859474 (SRR2179846, SRR2179847, SRR2179848, and SRR2179849)/GSM1859475 (SRR2179850, SRR2179851, SRR2179852, and SRR2179853) (wild-type/*ros1* mutant) and GSM819122/GSM819123/GSM819128/GSM819129 (wild-type/*rdd* mutant). The mRNA-Seq data from the wild-type, *ros1*, and *rdd* mutants were downloaded from the GEO database. Their GEO accession numbers are GSM1585887/GSM1585888/GSM1585889/GSM1585899/GSM15 85900/GSM1585901 (wild-type/*ros1* mutant). The *rdd* mRNA-Seq data were retrieved from the NCBI SRA database,^2^ whose accession numbers are SRR013411/SRR013412/SRR013413/SRR013414/SRR013415/SR R013416/SRR013426/SRR013427/SRR013428/SRR013429 (wild-type//*rdd* mutant).

### Processing of *Arabidopsis* BS-Seq Data

The SRA-formatted BS-Seq data were changed into the FASTQ format, and their sequencing quality was then evaluated. The adapters for sequencing were removed, and the low-quality bases were deleted. The clean BS-Seq reads were mapped to the TAIR10 genome (v36) with Bismark (v0.16.3) (Krueger and Andrews, 2011), allowing one base mismatch, and the unique paired-end reads were obtained for next analysis. To ensure dependable sequencing sites, cytosines covered by at least four reads were selected.

### Methylation Analysis of *Arabidopsis NLR* Genes

*Arabidopsis* typical *NLR* genes encoding both NB and LRR domains were selected for further analysis (Meyers et al., 2003). The gene body region (GBR) (transcribed region) covers the genomic region from the transcription start site to the end site. The chromosomal coordinates of *Arabidopsis NLR* GBRs and 200- and 500-bp regions upstream of the transcription start sites were determined with the TAIR10 annotation file^3^ by custom Perl scripts (Supplementary Table S1). The cytosine methylation levels were calculated as described previously (Kong et al., 2018).

### Processing of *Arabidopsis* mRNA-Seq Data

Possible adaptor sequences were cleaned from all the sequences before the reads were mapped to the *Arabidopsis* reference genome sequence, and the reads for which more than 50% of the bases had a low-quality value (≤5) were discarded. Then, the filtered reads were mapped through TopHat (v. 2.1.1) (Trapnell et al., 2012) to the TAIR10 genome sequence. The abundance of the *Arabidopsis* gene transcripts was determined and normalized with FPKM, that is, the expected fragments per kilobase of a transcript per million fragments sequenced, by Cufflinks software (v.2.2.1) (Trapnell et al., 2010, 2012).

HTSeq^4^ was used to measure the raw counts for all *Arabidopsis* genes determined through the TAIR10 annotation for coding genes (Anders et al., 2015). Then, the Cuffdiff program in the Cufflinks package (v2.2.1) was adopted to generate the differential expression data from these counts. The differentially expressed genes in each compared group were identified by the cutoff value of a more than twofold change and an adjusted *p*-value or FDR (false discovery rate) threshold ≤0.05.

### RNA Isolation and Real-Time Polymerase Chain Reaction Analysis

Total *Arabidopsis* RNAs were extracted from 2-week-old seedlings by TRIpure reagent (Aidlab Biotech, Beijing, China), and the possible contaminating DNAs were digested with DNase I (TransGen, Beijing, China). Two micrograms of total RNA was used for first-strand cDNA synthesis with the PrimeScript RT reagent kit (Takara, Dalian, China) according to the manufacturer’s instructions. The cDNA reaction mixtures were then diluted fivefold. In a 20-μL polymerase chain reaction (PCR) mixture, 1 μL of the diluted cDNA solution was pipetted into a tube as the template. *Arabidopsis ACTIN2* was used as an internal control. Program Premier 3 (Koressaar and Remm, 2007; Untergasser et al., 2012) was used to design the quantitative reverse transcriptase (qRT)–PCR primers (Supplementary Table S2). Quantitative reverse transcriptase–PCR was performed using the ABI 7500 Real Time PCR System (ABI, Carlsbad, CA, United States) with TransStart Top Green qPCR SuperMix (TransGen, Beijing, China). Three independent PCR analyses were carried out. The relative transcript levels were determined by the comparative threshold cycle (Ct) method (Relative Quantification Getting Started Guide; ABI). The mean fold changes were calculated using Livak’s 2^–ΔΔ(*Ct*)^ method (Livak and Schmittgen, 2001).

## Results

### Set of *Arabidopsis NLR* Genes Targeted by DNA Demethylases

DNA methylation occurring in the UPRs and within the transcribed gene bodies was observed in the majority of *NLR* genes in wild-type *Arabidopsis* plants, and the average methylation level of CG sequence contexts was greatly higher than that of CHG and CHH sequence contexts (Kong et al., 2018). In this study, we examined the DNA methylation status of *NLR* genes in the *ros1* and *rdd* mutant backgrounds by analyzing the BS-Seq data available from both the mutants and the corresponding wild-type controls (Supplementary Tables S3, S4).

**FIGURE 1:**
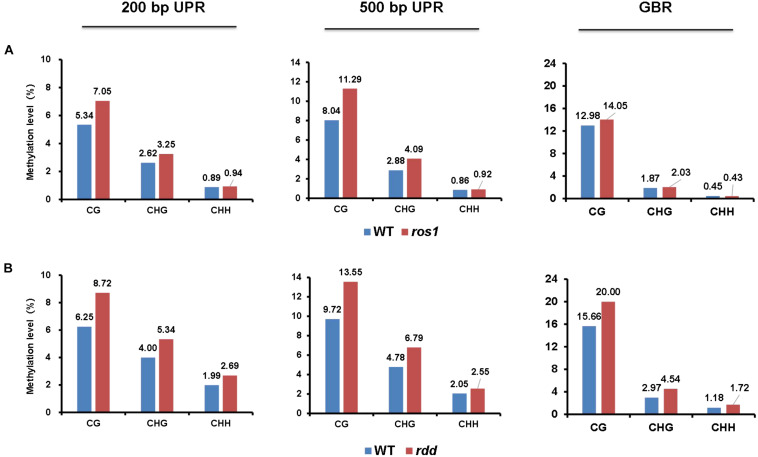
Average methylation level of *Arabidopsis NLR* genes in the wild-type (WT) and different demethylase mutant plants. **(A)** *ros1* mutant; **(B)** *rdd* mutant. UPR, upstream region; GBR, gene body region.

Our results demonstrated that for CG, CHG, and CHH sequence contexts, the average methylation levels in the 200- and 500-bp regions lying immediately upstream of transcriptional starting sites and of the entire transcribed gene bodies of the 144 *Arabidopsis NLR* genes were, in most situations, increased in *ros1* and *rdd* mutants relative to wild-type controls, indicating that the *NLR* genes in general are the targets of DNA demethylases (i.e., ROS1, DML2, and/or DML3) (Figure 1). In addition, the average methylation level of CG sequence contexts of the *Arabidopsis NLR* genes was clearly higher than the levels of CHG and CHH sequence contexts in both the *ros1* and *rdd* mutants (Figure 1).

**FIGURE 2:**
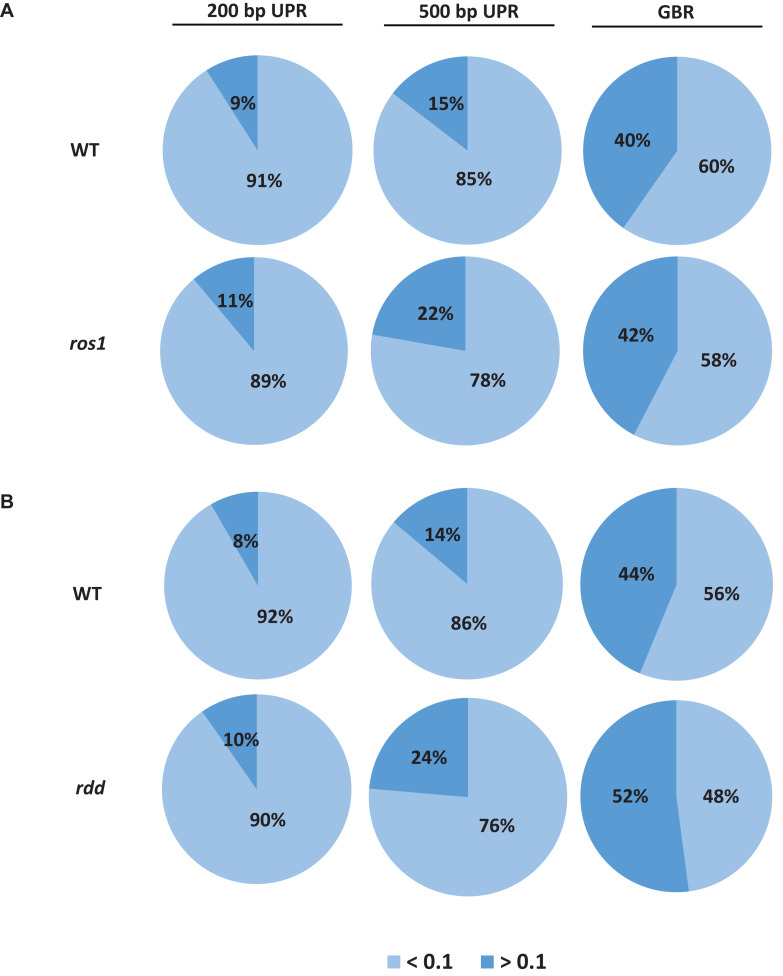
Distribution of the average methylation level of *Arabidopsis NLR* genes in the WT and different mutants defective in DNA demethylases. **(A)** *ros1* mutant; **(B)** *rdd* mutant. UPR, upstream region; GBR, gene body region.

Because the average methylation level of the CG sequence contexts was significantly higher than those of the CHG and CHH sequence contexts in the *ros1* and *rdd* mutants, the 144 *Arabidopsis NLR* genes were classified into two groups on the basis of their CG methylation levels: group 1 (>0.1), whose methylation level is greater than 0.1, and group 2 (<0.1), whose methylation level is less than 0.1. The results demonstrated that the CG methylation levels of these *NLR* genes in *ros1* and *rdd* at the 200-bp UPR, 500-bp UPR, and GBR are all increased because the proportions of group 1 in both mutants at the three regions increase consistently compared to those in wild-type controls. For example, the proportions of group 1 at 500-bp UPR in *ros1* and *rdd* were 22 and 24% versus 15 and 14% in the corresponding wild-type controls (Figure 2). By comparison, the proportions of group two at three such regions in both mutants were decreased overall (Figure 2). It is worth noting that the increase of proportions at 500-bp UPR is more dramatic in both *ros1* and *rdd* mutants than at the other two regions in their respective wild-type controls (Figure 2). Thus, these data collectively suggest that the mutations of the DNA demethylases generally lead to hypermethylation at the 200-bp UPRs, 500-bp UPRs, and GBRs of these *NLR* genes, and the 500-bp UPRs gain a higher level of methylation than the other two regions.

**FIGURE 3:**
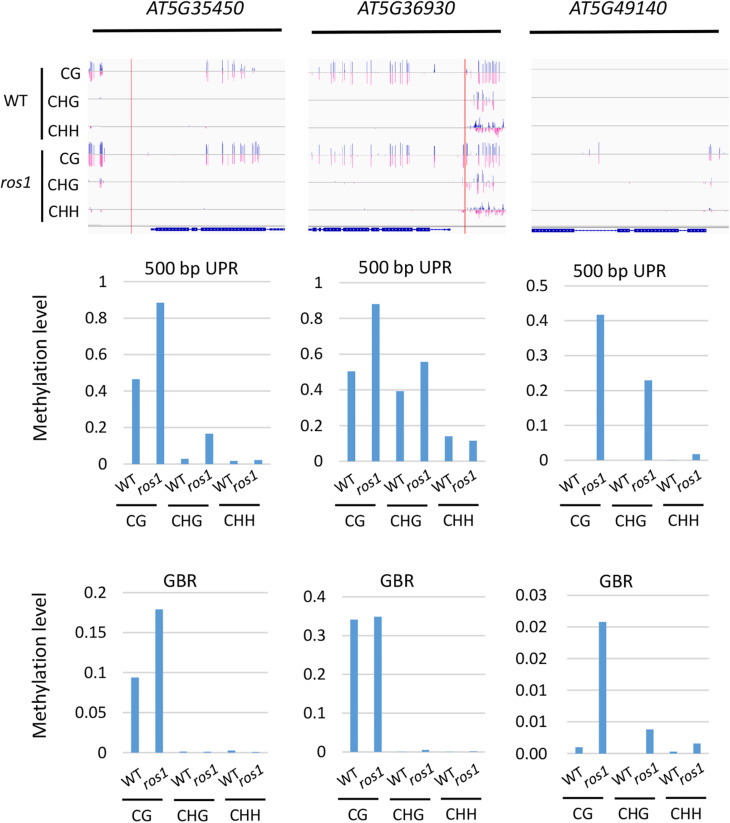
Methylated *NLR* genes from *Arabidopsis* WT (Col-0) and *ros1* mutants. For each *NLR* gene, the DNA methylation patterns in the WT and *ros1* mutant are shown in the upper panel, and its DNA methylation levels are illustrated in the lower panels. The red rectangle indicates the 500-bp UPR.

To further determine which members of the *NLR* genes have undergone evident changes in DNA methylation level in *ros1* and *rdd* mutants, the CG methylation levels of all the *NLR* genes were analyzed. Our results demonstrated that there are 10 *NLR* genes in which CG methylation levels at the 200-bp UPRs are significantly different between *ros1* and wild-type plants (Supplementary Table S5); eight of them show at least a 10% increase in DNA methylation level in the *ros1* mutant compared with the wild-type control. For *AT5G49140*, *AT5G35450*, and *AT5G36930*, their CG methylation levels were more than 50% higher in the *ros1* mutant relative to the wild-type control (Figure 3 and Supplementary Table S5). In contrast, two genes, that is, *AT4G09430* and *AT2G17050*, exhibited decreased CG methylation levels in the *ros1* mutant, and notably, the proportion of CG methylation of *AT2G17050* decreased from 77.27% to zero (Supplementary Table S5).

For 500-bp UPRs, all 18 examined genes but one (*AT1G12280*) in *ros1* showed no less than 10% increase in CG methylation level compared to the wild-type control (Supplementary Table S5). Two genes, *AT5G35450* and *AT5G49140* in *ros1*, display a methylation increase of greater than 40% relative to wild type (Supplementary Table S5 and Figure 3). It should be noted that there are four genes (i.e., *AT5G49140*, *AT4G27190*, *AT1G31540*, and *AT1G59780*) with no methylation at such regions in the wild-type control, showing increased methylation at least 20% in the *ros1* mutant. For the methylation status of GBRs, it appears that there are no obvious differences in DNA methylation levels between *ros1* and the wild-type control because the maximum difference is less than 9% as exemplified by *AT5G35450*, suggesting that the transcribed gene bodies of such *NLRs* are not the main targets of ROS1 (Supplementary Table S5).

**FIGURE 4:**
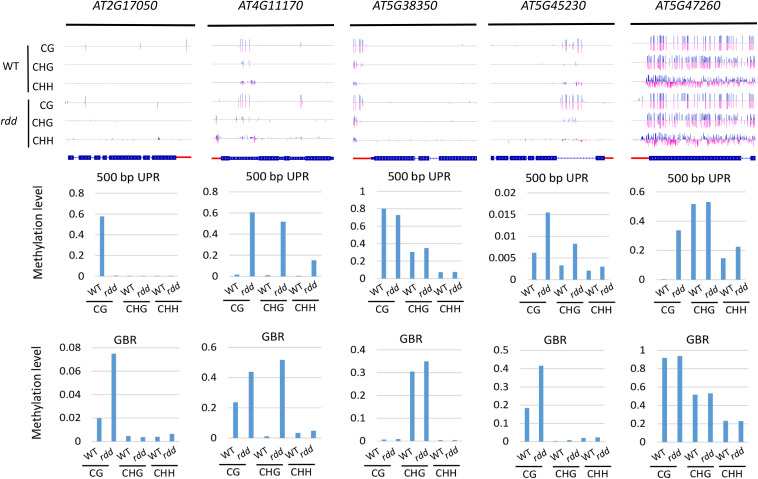
Methylated *NLR* genes from *Arabidopsis* WT and *rdd* mutants. For each *NLR* gene, the DNA methylation patterns in the WT and *rdd* mutant are shown in the upper panel, and its DNA methylation levels are illustrated in the lower panels. The red rectangle indicates the 500-bp UPR.

In the *rdd* mutant, the CG methylation levels in the 200-bp UPRs of eight of nine *Arabidopsis NLR* genes increased by more than 10% (Supplementary Table S6). It is worth noting that three genes (*AT5G47260*, *AT4G11170*, and *AT5G45240*) have notably low levels of DNA methylation in the wild-type control, whereas they show a substantial increase of more than 40% in methylation levels in the *rdd* mutant (Supplementary Table S6 and Figure 4).

**FIGURE 5:**
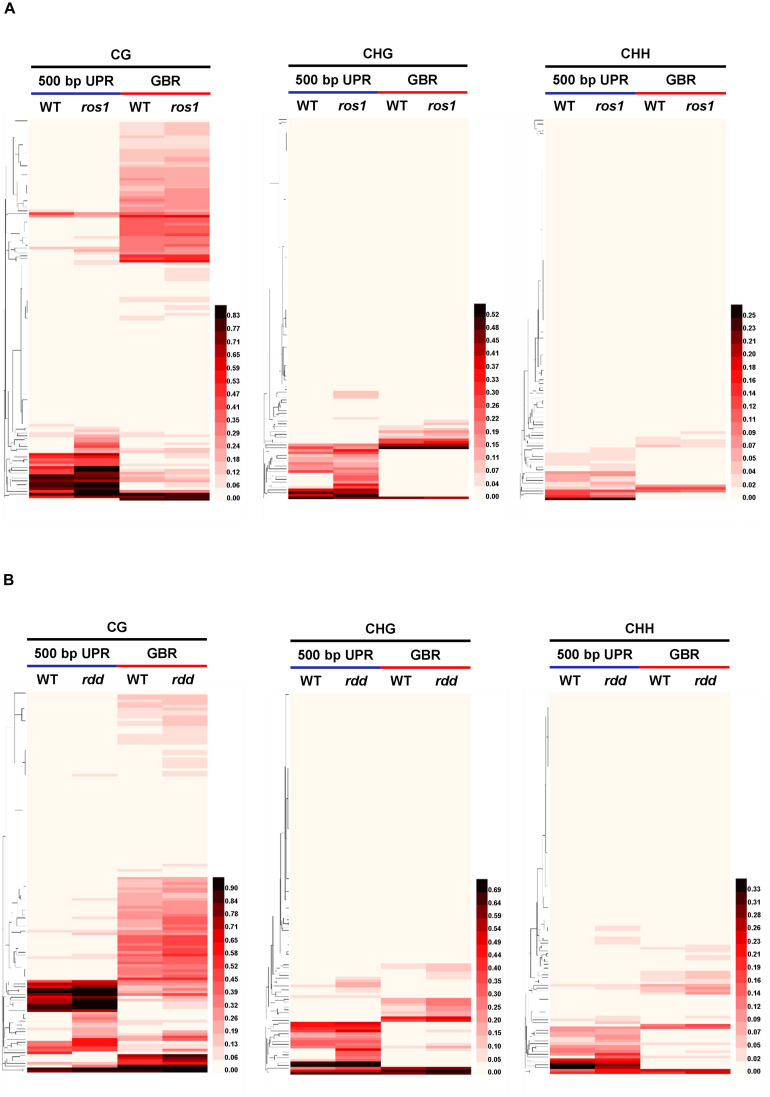
Heatmap of the methylation levels of 144 *Arabidopsis NLR* genes in diverse genotypes. **(A)** *NLR* genes in wild type and *ros1* mutant; **(B)** *NLR* genes in wild type and *rdd* mutant.

Within the 500-bp UPRs, there were 23 of 24 *NLR* genes, which all exhibited a growth of 10% in methylation levels, and five of these genes displayed a 30% increase in methylation levels in the *rdd* mutant compared to the wild-type control. In contrast, the methylation level of *AT2G17050* was reduced by approximately 57% in *rdd* (Supplementary Table S6). For GBRs, 23 of 43 examined *NLR* genes showed an increase in methylation levels by more than 10% in *rdd* compared to the wild-type control (Supplementary Table S6 and Figure 4). Notably, the increase in DNA methylation levels is generally larger within the 500-bp UPRs than the GBRs (Figures 5A,B). These data collectively indicate that triple mutations of ROS1, DML2, and DML3 lead to DNA hypermethylation within the promoters, as well as gene bodies of some specific *NLR* genes.

Our above analysis also revealed that the DNA methylation levels of different *NLR* genes in the same mutant were highly different (Figures 5A,B). For instance, in *ros1*, no DNA methylation was observed in the *AT1G58807*, *AT1G59124*, and *AT1G59218* genes (Supplementary Table S3). However, *AT1G27180* showed a lower level of DNA methylation in the *ros1* mutant than the corresponding wild-type control (Supplementary Table S3). Other *NLR* genes may be heavily methylated in *ros1* mutants. For example, *AT1G58602* had a CG methylation level of 52.23% and a CHG methylation level of 27.14% in its transcribed gene body in *ros1*; *AT3G46710* had 87.25% of CG methylation level and 15.04% of CHG methylation level in the upstream 500-bp region; *AT4G09360* had a CG methylation level of 86.25% and a CHG methylation level of 21.43% in the upstream 500-bp region, and its CG and CHG methylation levels within the transcribed region were 77.79 and 41.23%, respectively; in the upstream 500-bp region of *AT4G19520*, the CG methylation level was as high as 87.25%, and the CHG methylation level was 15.04%; for *AT5G36930*, the CG and CHG methylation levels in the upstream 500-bp region were as high as 88.03 and 55.63%, respectively (Supplementary Table S3). In the *ros1* mutant, unexpectedly, in the 200-bp UPR of *AT2G17050*, the CG methylation level was 77.27% lower than that of the wild-type control (Supplementary Table S3).

In the *rdd* mutant, a similar methylation profile exists (Figure 5B). Three genes, *AT1G58807*, *AT1G59124*, and *AT1G59218*, did not exhibit DNA methylation, whereas *AT1G12210*, *AT1G27180*, *AT1G56540*, and *AT5G46260* were less highly methylated, but *AT3G46710*, *AT4G09360*, *AT4G19520*, and *AT5G36930* were highly methylated at their CG and CHG sites (Supplementary Table S4). In this mutant, compared to the wild-type plants, the methylation levels of CG, CHG, and CHH sites in the upstream 200-bp region of *AT5G45240* were increased by 42.47, 29.56, and 16.66%, respectively, whereas the CG methylation levels in the upstream 200-bp regions of *AT4G11170* and *AT5G47260* were 59.71 and 62.19% higher than those of the wild-type plants, respectively (Supplementary Table S4).

**FIGURE 6:**
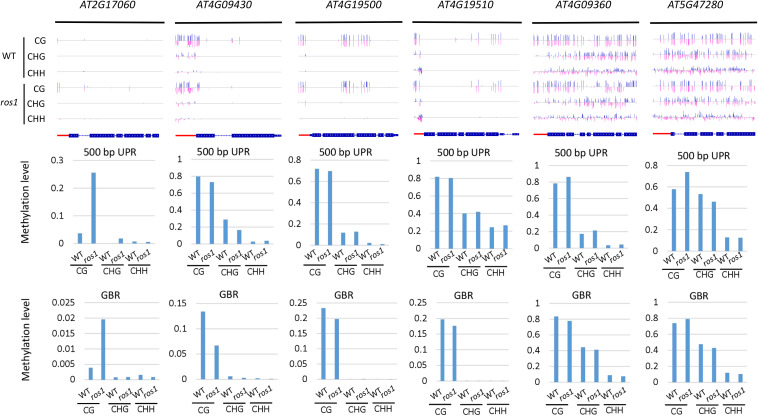
Highly methylated *NLR* genes from *Arabidopsis* WT and *ros1* mutants. For each *NLR* gene, the DNA methylation patterns in the WT and *ros1* mutant are shown in the upper panel, and its DNA methylation levels are illustrated in the lower panels. The red rectangle indicates the 500-bp UPR.

**FIGURE 7:**
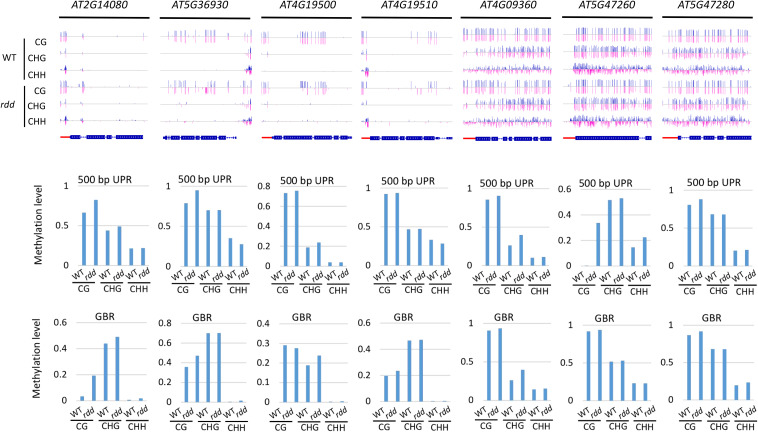
Highly methylated *NLR* genes from *Arabidopsis* WT and *rdd* mutants. For each *NLR* gene, the DNA methylation patterns in the WT and *rdd* mutant are shown in the upper panel, and its DNA methylation levels are illustrated in the lower panels. The red rectangle indicates the 500-bp UPR.

DNA demethylases play an important role in inhibiting the hypermethylation of endogenous genes in plants. However, this study demonstrated that some *Arabidopsis NLR* genes show high DNA methylation not only in *ros1* and *rdd* mutants but also in wild-type plants (Figures 5A,B). The DNA methylation of these genes was found to be similar between the wild-type and mutant plants. For example, *AT4G09360* and *AT5G47280* were highly methylated in the UPRs and transcribed gene bodies in both the *ros1* mutants and the wild-type plants (Figure 6 and Supplementary Table S3). In the UPRs of *AT4G19500* and *AT4G19510*, three cytosine sequence contexts were highly modified by DNA methylation in the wild-type and *ros1* mutant plants, and CG methylation was observed within their transcribed regions (Figure 6 and Supplementary Table S3). The other two genes, *AT2G17060* and *AT4G09430*, were hypermethylated primarily in their UPRs in the *ros1* mutants and wild-type plants (Figure 6 and Supplementary Table S3). In the wild-type and *rdd* mutant, *AT4G09360*, *AT5G47260*, and *AT5G47280* were heavily methylated in the three cytosine sequence contexts of the upstream and transcribed regions (Figure 7 and Supplementary Table S4); *AT5G36930* was also clearly modified by DNA methylation, and the three cytosine sequence contexts of its UPR were significantly modified by DNA methylation, but CG methylation was mainly found within its transcribed region (Figure 7 and Supplementary Table S4). Interestingly, *AT4G09360* and *AT5G47280* were hypermethylated in both *ros1* and *rdd*, as well as their respective wild-type plants (Figures 6, 7 and Supplementary Tables S3, S4). In addition, *AT4G19500* and *AT4G19510* were the same as these two genes, but their methylation levels were considerably lower in the extent of modification (Figures 6, 7 and Supplementary Tables S3, S4). The maintenance of heavy DNA methylation within these genes in wild-type plants suggests that DNA demethylases have little effect on them and that hypermethylation plays a critical role in their functions.

### Transcriptional Activities of *Arabidopsis NLR* Genes in Wild-Type Plants and Various DNA Demethylase Mutants

It has been reported that there is a close relationship between DNA methylation and the transcriptional activity of a gene (Zilberman et al., 2007). The expression of *NLR* genes in *Arabidopsis* and soybean has also been shown to be regulated by their DNA methylation levels (Kong et al., 2018; Richard et al., 2018). To determine whether the mutations of the DNA demethylases affect the transcriptional activities of the *Arabidopsis NLR* genes, this study analyzed the available mRNA-Seq data from the *Arabidopsis ros1* and *rdd* mutants and their respective wild-type controls to examine the transcriptional activities of the *Arabidopsis NLR* genes.

**FIGURE 8:**
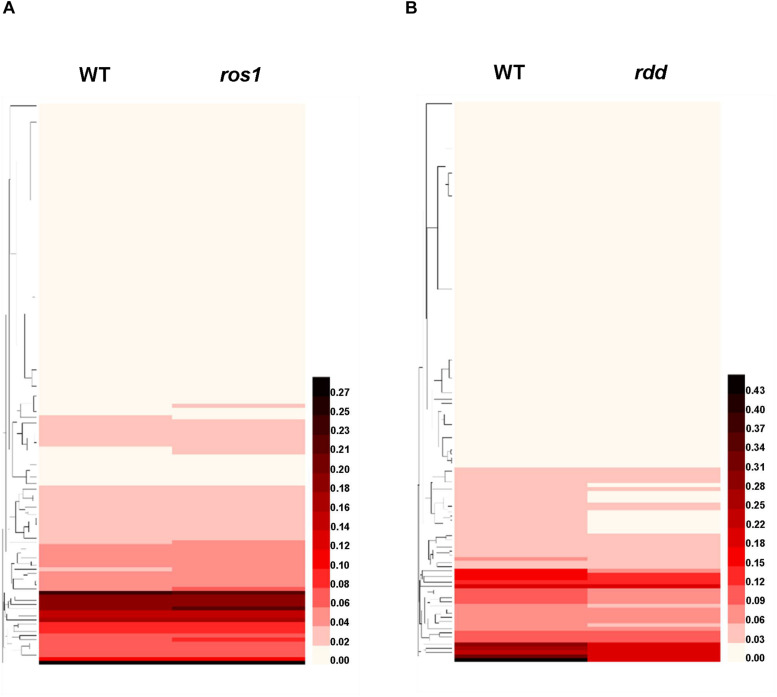
Heatmap of the transcriptional levels of 144 *Arabidopsis NLR* genes in diverse genotypes. **(A)** *NLR* genes in wild type and *ros1* mutant; **(B)** *NLR* genes in wild type and *rdd* mutant.

An overall analysis of the transcriptional level of the *NLR* genes in the wild-type and mutants indicated that the expression levels of most *NLR* genes were very low in both wild-type plants and the mutants (Figure 8). However, most of the *NLR* genes with relatively high transcriptional activity in the wild-type plants showed a slightly higher expression level after the mutation of ROS1 (Figure 8A), whereas most of those *NLR* genes with relatively high expression levels in the wild-type plants demonstrated reduced expression in the *rdd* mutant (Figure 8B). Specifically, our analysis revealed that there are 43 transcribed *NLR* genes with the value of at least one FPKM in the wild-type plants or *ros1* mutants, and their ratios of FPKM values in *ros1* to the wild-type plants are ≥1.1 or ≤0.9 (Supplementary Table S7). Among these genes, the FPKM values of 38 *NLR* genes increased, and those of five *NLR* genes decreased, in *ros1* relative to the wild-type plants (Supplementary Table S7). It is noted that the FPKM value of *AT4G19520* in *ros1* was even 1.97 times the value of the gene in the wild-type plants, suggesting that the mutation of ROS1 contributes to the transcription of such genes (Supplementary Table S7). However, there are five genes (i.e., *AT1G58602*, *AT1G10920*, *AT1G63750*, *AT1G62630*, and *AT1G59620*) that all have FPKM values of less than 1.0, indicating that these five genes are downregulated in the *ros1* mutant (Supplementary Table S7).

In the wild-type or *rdd* mutant plants, 64 *NLR* genes were found to be expressed with the value of at least one FPKM, and the ratios of the FPKM values were ≥1.1 or ≤0.9 (Supplementary Table S8). Only the ratios of FPKM values of *AT1G12280*, *AT1G61180*, and *AT4G19520* were over 1.1 in the *rdd* mutants, whereas the ratios of the other 61 *NLR* genes were all less than 0.9 (Supplementary Table S8). We also observed that the change in the transcriptional level of some *NLR* genes was inconsistent in *ros1* and *rdd* mutants; however, the transcriptional levels of *AT1G12280*, *AT1G61180*, and *AT4G19520* were higher in both *ros1* and *rdd* mutants than in the wild-type plants. In contrast, the transcriptional levels of *AT1G58602*, *AT1G59620*, and *AT1G62630* were lower in both *ros1* and *rdd* mutants than in the wild-type plants (Supplementary Table S9). This finding suggests that the transcriptional activities of these genes were likely to be regulated by DNA demethylases.

**FIGURE 9:**
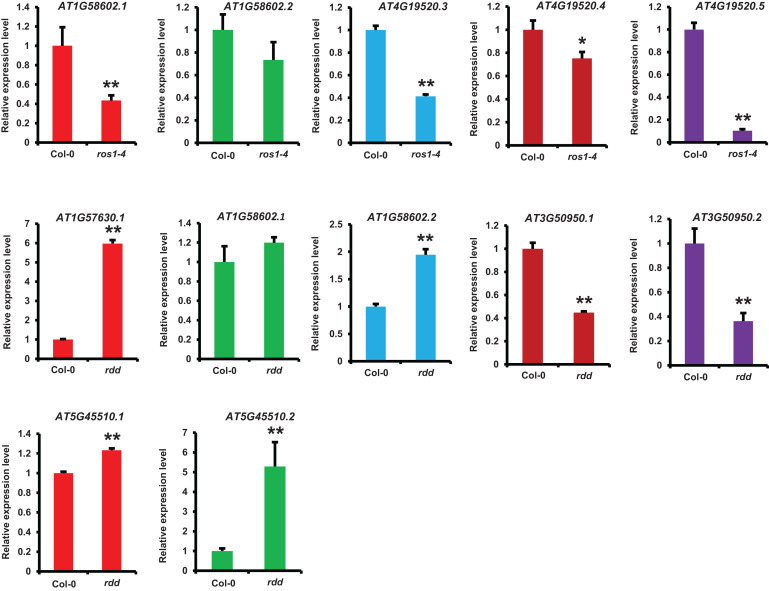
Detection of the expression of *NLR* genes in different *Arabidopsis* genotypes by qRT-PCR. *ACTIN2* was used as an internal control. Error bars represent the standard deviation from three technical replicates. One-way analysis of variance (ANOVA) was applied to examine gene expression differences between the WT and each mutant. *Statistical significance (*p* ≤ 0.05) and **high statistical significance (*p* ≤ 0.01).

We identified the differentially expressed *NLR* genes between *ros1* or *rdd* and wild-type plants by analyzing their mRNA-Seq data and then verified some identified *NLR* genes using real-time qRT-PCR. Five selected *NLR* genes were confirmed to be differentially expressed between the mutants and the wild-type plants (Figure 9). The expression levels of 10 transcripts encoded by these five *NLR* genes were detected in *ros1* and *rdd* mutants. The results demonstrated that the expression levels of *AT1G58602.1*, *AT4G19520.3*, *AT4G19520.4*, and *AT4G19520.5* were reduced in the *ros1* mutant relative to Col-0 (Figure 8). Among these genes, *AT4G19520.5* expression was notably reduced in the *ros1* mutant (Figure 8). In *rdd* mutants, *AT3G50950.1* and *AT3G50950.2* were detected to be reduced in expression compared with Col-0 (Figure 8). In contrast, in *rdd* mutants, *AT1G57630.1*, *AT1G58602.2*, and *AT5G45510.1* were upregulated relative to Col-0 (Figure 8). Thus, some *NLR* genes are suggested to be regulated by DNA demethylases.

## Discussion

### Methylation Patterns of Some *NLR* Genes in *Arabidopsis* Are Shaped by Both DNA Methyltransferases and Demethylases

The DNA methylation patterns of some plant genes can be established and maintained by DNA methyltransferases, whereas those of others are jointly shaped by both methyltransferases and demethylases (Zhu, 2009). Our analyses revealed that the average methylation levels of the CG, CHG, and CHH sequence contexts in the 500-bp UPRs and transcribed gene bodies of *Arabidopsis NLR* genes varied in different DNA demethylase mutants. In *ros1* and *rdd* mutants, the average methylation levels of the three cytosine sequence contexts within the *NLR* genes were increased, but to a different extent. The average CG methylation levels within the *NLR* genes were higher than the average CHG and CHH methylation levels in the *ros1* and *rdd* mutants. It has been shown that most of the CG sites of some transposons and other genes are highly methylated in wild-type plants, whereas many CHG and CHH sites of the transposons and genes are methylated slightly or are even completely unmethylated; however, in *ros1*, these CHG and CHH sites are heavily methylated (Zhu et al., 2007). Similar to this result, higher levels of CHG and CHH methylation were observed within the *NLR* genes in *ros1* and *rdd* mutants than in the wild-type plants. Further analysis revealed increased *NLR* genes with a CG methylation level higher than 10% and decreased *NLR* genes with a CG methylation level of less than 10% in *ros1* and *rdd* mutants.

Among all the known demethylases in *Arabidopsis*, ROS1 is regarded as the predominant DNA demethylase in vegetative tissues (Tang et al., 2016). However, mutations of DML2 and/or DML3 were observed to cause the hypermethylation of unmethylated or weakly methylated cytosine residues in wild-type plants (Ortega-Galisteo et al., 2008). Additionally, the heavily methylated cytosines in wild-type plants were shown to be hypomethylated in the *dlm2* and/or *dml3* mutants (Ortega-Galisteo et al., 2008). Additionally, most of the hypermethylated loci in *ros1-4* were found to overlap with those in the *rdd* mutant (Qian et al., 2012). Thus, ROS1, DML2, and DML3 have their own distinct targets, although they overlap at some loci. We found that eight *NLR* genes (*AT1G56540*, *AT3G04220*, *AT4G09430*, *AT5G40100*, *AT5G45510*, *AT5G46260*, *AT5G47280*, and *AT5G49140*) showed elevated or reduced methylation levels by at least 10% within their 200- or 500-bp UPRs in *ros1* mutants but not *rdd* mutants (Supplementary Table S10). Similar changes were also observed in the other eleven *NLR* genes (*AT1G61180*, *AT3G07040*, *AT3G46530*, *AT4G16960*, *AT4G33300*, *AT5G38350*, *AT5G40060*, *AT5G45240*, *AT5G46490*, *AT5G47250*, and *AT5G47260*) in the *rdd* mutant (Supplementary Table S10). On the other hand, there are 14 *NLR* genes whose alterations in CG methylation level by at least 10% within the UPRs occurred in *ros1* mutants, as well as *rdd* mutants (Supplementary Table S10). Within the transcribed regions, 15 *NLR* genes displayed an increased CG methylation level by at least 5% in the *ros1* mutant (Supplementary Table S11). However, 41 *NLR* genes showed an increase in CG methylation level by at least 5% in the *rdd* mutant, and 23 of them showed an increase up to greater than 10% (Supplementary Table S11). Among these genes, five *NLR* genes, which are *AT5G45230*, *AT4G09430*, *AT4G08450*, *AT1G53350*, and *AT5G05400*, displayed altered methylation in both *ros1* and *rdd* mutants (Supplementary Table S11). Additionally, *AT4G09360* and *AT4G09430* showed decreased CG methylation in the *ros1* mutant; *AT3G04220* and *AT4G19050* showed a decrease in the *rdd* mutant (Supplementary Table S11). Therefore, each DNA demethylase exerts a specific effect on the DNA methylation of the *NLR* genes. Similarly, the methylation levels within 7 of 14 loci in each single mutant were observed to be considerably less than in the *rdd* triple mutant, indicating that all the DML enzymes jointly demethylate these loci, whereas some other loci were found to be demethylated by a single DML (Penterman et al., 2007). Hence, these three glycosylases function with partial redundancy. In this study, ROS1 mutation does not cause an increase in DNA methylation at all *NLR* genes, even hypomethylation at some *NLR* genes can be observed, also suggesting that DML2 and/or DML3 are able to compensate for ROS1 loss at some targets. It has been reported that the DNA methylation patterns of many *Arabidopsis NLR* genes are regulated by different DNA methyltransferases (Kong et al., 2018). Taken together, these results indicate that the methylation patterns of many *NLR* genes in *Arabidopsis* are regulated not only by DNA methyltransferases but also by DNA demethylases.

### DNA Demethylases Mediate the Transcriptional Activities of *NLR* Genes in *Arabidopsis thaliana*

It has been revealed that the DNA methylation levels of some genes in *Arabidopsis* are closely related to their transcriptional activities (Penterman et al., 2007). The mutation of ROS1 leads to increased DNA methylation and decreased expression in some *Arabidopsis* genomic loci (Zhu et al., 2007). Another study has shown that *Arabidopsis* DNA demethylases, including ROS1, DML2, and DML3, are able to modulate the transcriptional activity of many stress response genes, and these stress response genes are repressed in the *rdd* mutant (Le et al., 2014). In this study, we show that the transcriptional levels of some *NLR* genes are higher in different mutants defective in DNA demethylase than in the wild-type controls, whereas the levels of other *NLR* genes are lower in diverse DNA demethylase–defective mutants than in their wild-type controls.

We found that 28 *NLR* genes were upregulated in *ros1* but downregulated in *rdd* mutants in comparison to the wild-type controls, three *NLR* genes were upregulated in both *ros1* and *rdd* mutants, and one *NLR* gene (*AT1G62630*) was downregulated in both *ros1* and *rdd* mutants (Supplementary Table S12). We also observed that nine *NLR* genes were upregulated and two *NLR* genes (*AT1G10920* and *AT1G63750*) were downregulated only in *ros1* but *rdd* mutants (Supplementary Table S12). In addition, we discovered that 32 *NLR* genes were repressed in the *rdd* mutant (Supplementary Table S12). The *rdd* mutant was shown to exhibit increased susceptibility to *F. oxysporum*, and the transcriptional activities of *AT1G58602* and *AT4G09420* were detected to be downregulated (Le et al., 2014). Thus, the three demethylases may play partially redundant roles, and DML2 and/or DML3 can partially compensate some *NLR* genes for the loss of function of ROS1. On the other hand, the transcriptional activities of many *NLR* genes in *Arabidopsis* are mediated by different DNA demethylases, and the transcriptional activity varied among different *NLR* genes when the DNA demethylases were mutated.

Our qRT-PCR results further confirmed that some transcripts encoded by *Arabidopsis NLR* genes were increased or decreased at the transcriptional level in the mutants defective in DNA demethylases. Therefore, it is important and meaningful to reveal the mechanisms by which DNA demethylases modulate the expression of *Arabidopsis NLR* genes.

### Relationships Between Methylation and Transcription of *Arabidopsis NLR* Genes

It was reported that only 182 genes demonstrated altered methylation (Penterman et al., 2007), and 167 genes presented differential expression (Lister et al., 2008) in the *rdd* mutant compared to wild-type plants. Therefore, changes in DNA methylation or gene expression are limited in the *rdd* mutant compared to wild-type plants. In another study, 348 genes were observed to be differentially expressed (Le et al., 2014). In their studies, the differentially expressed genes seldom overlapped with the differentially methylated genes (Le et al., 2014). We also found little overlap in a few *NLR* genes. For instance, three *NLR* genes (*AT1G31540*, *AT5G35450*, and *AT5G44870*) showed increased CG methylation within 500-bp UPRs and elevated transcriptional activity in the *ros1* mutant compared to wild-type plants, whereas *AT1G12280* showed decreased CG methylation and elevated expression when ROS1 was mutated (Supplementary Table S13). In the *rdd* mutant, 11 *NLR* genes showed CG hypermethylation within the 500-bp UPRs, nine of which were downregulated compared to wild-type plants, whereas *AT1G12280* and *AT1G61180* were upregulated (Supplementary Table S14), suggesting that a close link exists between CG hypermethylation within UPRs and the expression of these *NLR* genes in the *rdd* mutant. Interestingly, a similar link occurs between CG hypermethylation within gene transcribed regions and the differential expression of 11 *NLR* genes in the *rdd* mutant (Supplementary Table S14). Of the 11 *NLR* genes, with the exception of *AT4G19520*, 10 were downregulated in the *rdd* mutant compared to wild-type plants. It is worth noting that *AT4G33300*, *AT5G36930*, and *AT5G44870* showed increased CG methylation within 500-bp UPRs and GBRs and downregulated expression in the *rdd* mutant compared to wild-type plants (Supplementary Table S14), indicating a negative connection between CG hypermethylation and their downregulated expression. Nevertheless, many *NLR* genes have no direct link between their changes in methylation status and transcriptional activity. A previous study also suggested the regulation of defense genes by DNA methylation not only based on *cis*-acting modes but also in *trans*, as well as the global influence of DNA demethylation on the activation of the defense-associated transcriptome through primarily *trans*-regulatory mechanisms (Lopez Sanchez et al., 2016).

## Conclusion

In this study, we show that some *Arabidopsis NLR* genes can be demethylated by ROS1, DML2, and DML3 within their upstream and transcribed regions. We revealed that the loss of functions of the demethylases leads to obvious changes in DNA methylation levels within some members of *Arabidopsis NLR* genes. We found that demethylases have no effects on the DNA methylation status of some *Arabidopsis NLR* genes. We demonstrated that some *Arabidopsis NLR* genes were regulated by the DNA demethylases ROS1, DML2, and/or DML3. This study will provide a reference for future research into the expression of *Arabidopsis NLR* genes.

## Data Availability Statement

All datasets generated for this study are included in the article/Supplementary Material.

## Author Contributions

WK conceived the project, designed study, interpreted the data, and wrote the manuscript. WK, HL, and AL supervised the study design. HL provided the plant materials and edited the manuscript. XX, L-WL, SZ, and LD conducted the bioinformatic analyses of the DNA methylome, transcriptome and statistical analyses of the experimental data. QW carried out the qRT-PCR assays. All authors approved the final manuscript.

## Conflict of Interest

The authors declare that the research was conducted in the absence of any commercial or financial relationships that could be construed as a potential conflict of interest.

## Footnotes

1 https://www.ncbi.nlm.nih.gov/gds

2 https://www.ncbi.nlm.nih.gov/sra

3 https://www.arabidopsis.org

4 https://htseq.readthedocs.io/en/release_0.10.0/
