# Supplementary material for: Impact of DNA Demethylases on the DNA Methylation and Transcription of Arabidopsis NLR Genes
Source: Front Genet. 2020 May 26;11:460. doi: 10.3389/fgene.2020.00460 (PMC7264425; doi:10.3389/fgene.2020.00460)
Supplement: Supplementary file 2 [file Table_2.DOCX]

**TABLE S2 |** Primer sequences used for qRT-PCR assays of Arabidopsis *NLR* genes. The primers were designed using the Primer Premier 5.0.

| **Primer Name** | **Sequence (5'→3')** |
| --- | --- |
| AT1G57630.1-F | CGGGGAAGATGTACGCAAAG |
| AT1G57630.1-R | TGGAGAGCAAGACAAGAGCA |
| AT1G58602.1-F | AGAGCCAGGAGTCTCATAAG |
| AT1G58602.1-R | GTAGCTTCAACCTCGTATCA |
| AT1G58602.2-F | AGTCTGAAGCAGAGAGACAT |
| AT1G58602.2-R | AGTCTTGAGTAGTAGTAGTC |
| AT3G50950.1-F | CGTAGTCGGCAGCAGTAACC |
| AT3G50950.1-R | GTTACAACAGCGTCCACCAT |
| AT3G50950.2-F | ACGAGTAAGGTTGACTCTTG |
| AT3G50950.2-R | ACCATTTACTCAGACCCTGC |
| AT4G19520.3-F | AGTGATGCCTATTTTCTACA |
| AT4G19520.3-R | TGCATCCGATGCCCTACCAA |
| AT4G19520.4-F | AATTACATATAAATACTCAG |
| AT4G19520.4-R | TTAGGGACTAATATCTTGAA |
| AT4G19520.5-F | TGAGCGACAAAGATGGAGTC |
| AT4G19520.5-R | GATGATATTAGCTTCTTCCG |
| AT5G45510.1-F | TATTGCTAAGTTAGAGAATC |
| AT5G45510.1-R | CTGTGGTCAACCATTACGTA |
| AT5G45510.2-F | GATGCCTGCCAGCCTACCAG |
| AT5G45510.2-R | CTGCAAATACACGCAGTGTA |
| AT3G18780.1-F  (*ACTIN2*) | GCCATCCAAGCTGTTCTCTC |
| AT3G18780.1-R  (*ACTIN2*) | GCTCGTAGTCAACAGCAACAA |
