# Supplementary material for: Impact of DNA Demethylases on the DNA Methylation and Transcription of Arabidopsis NLR Genes
Source: Front Genet. 2020 May 26;11:460. doi: 10.3389/fgene.2020.00460 (PMC7264425; doi:10.3389/fgene.2020.00460)
Supplement: Supplementary file 5 [file Table_5.DOCX]

**TABLE S5 |** CG methylation of some Arabidopsis *NLR* genes from wild-type (Col-0) and *ros1* mutants. Only reads mapping uniquely to the Arabidopsis nuclear genome were used, and cytosines covered by at least 4 reads were considered in this analysis.

| **Gene ID** | **200 bp UPR (%)** | | | **Gene ID** | **500 bp UPR (%)** | | | **Gene ID** | **GBR (%)** | | |
| --- | --- | --- | --- | --- | --- | --- | --- | --- | --- | --- | --- |
|  | **WT** | ***ros1*** | **ΔML** |  | **WT** | ***ros1*** | **ΔML** |  | **WT** | ***ros1*** | **ΔML** |
| *AT5G49140* | 0 | 58.65 | 58.65 | *AT5G35450* | 46.49 | 88.48 | 41.99 | *AT5G35450* | 9.4 | 17.9 | 8.5 |
| *AT5G35450* | 34.42 | 87.98 | 53.56 | *AT5G49140* | 0 | 41.74 | 41.74 | *AT2G16870* | 17.35 | 25.64 | 8.29 |
| *AT5G36930* | 40 | 90.83 | 50.83 | *AT4G27190* | 0 | 37.98 | 37.98 | *AT5G44510* | 54.58 | 62.82 | 8.24 |
| *AT3G46710* | 42.76 | 88.11 | 45.35 | *AT5G36930* | 50.36 | 88.03 | 37.67 | *AT5G05400* | 9.43 | 17.56 | 8.13 |
| *AT4G11170* | 0 | 27.69 | 27.69 | *AT5G17890* | 44.35 | 73.85 | 29.5 | *AT4G36140* | 35.53 | 43.32 | 7.79 |
| *AT5G44870* | 58.25 | 77.86 | 19.61 | *AT1G31540* | 0 | 28.91 | 28.91 | *AT1G15890* | 12.01 | 19.66 | 7.65 |
| *AT3G04220* | 0 | 16 | 16 | *AT1G59780* | 0 | 28.57 | 28.57 | *AT5G46450* | 18.96 | 26.57 | 7.61 |
| *AT1G59780* | 0 | 11.52 | 11.52 | *AT3G46710* | 58.91 | 87.25 | 28.34 | *AT5G17970* | 0.26 | 7.08 | 6.82 |
| *AT4G09430* | 81.46 | 69.17 | -12.29 | *AT2G17060* | 3.68 | 25.62 | 21.94 | *AT1G53350* | 13.67 | 20.2 | 6.53 |
| *AT2G17050* | 77.27 | 0 | -77.27 | *AT4G11170* | 0.85 | 21.82 | 20.97 | *AT1G58410* | 4.4 | 10.33 | 5.93 |
|  |  |  |  | *AT5G47280* | 57.87 | 73.94 | 16.07 | *AT1G61310* | 0.12 | 5.94 | 5.82 |
|  |  |  |  | *AT5G45510* | 26.99 | 42.57 | 15.58 | *AT5G40910* | 35.39 | 40.63 | 5.24 |
|  |  |  |  | *AT5G46260* | 0.36 | 15.31 | 14.95 | *AT5G45230* | 9.3 | 14.52 | 5.22 |
|  |  |  |  | *AT5G40100* | 4.23 | 18.32 | 14.09 | *AT5G47280* | 73.99 | 79.11 | 5.12 |
|  |  |  |  | *AT2G14080* | 55.69 | 68.63 | 12.94 | *AT4G08450* | 16.81 | 21.92 | 5.11 |
|  |  |  |  | *AT5G51630* | 0.24 | 11.2 | 10.96 | *AT4G09360* | 83.46 | 77.79 | -5.67 |
|  |  |  |  | *AT1G56540* | 0.53 | 11.39 | 10.86 | *AT4G09430* | 13.41 | 6.69 | -6.72 |
|  |  |  |  | *AT1G12280* | 42.44 | 27.86 | -14.58 |  |  |  |  |

UPR: upstream region; GBR: gene body region; listed are the *NLR* genes whose methylation difference between the WT and mutants is > 10% within the UPR or > 5% within the GBR.
