# Supplementary material for: Impact of DNA Demethylases on the DNA Methylation and Transcription of Arabidopsis NLR Genes
Source: Front Genet. 2020 May 26;11:460. doi: 10.3389/fgene.2020.00460 (PMC7264425; doi:10.3389/fgene.2020.00460)
Supplement: Supplementary file 6 [file Table_6.DOCX]

**TABLE S6 |** CG methylation of some Arabidopsis *NLR* genes from wild-type (Col-0) and *rdd* mutants. Only reads mapping uniquely to the Arabidopsis nuclear genome were used, and cytosines covered by at least 4 reads were considered in this analysis.

| **Gene ID** | **200 bp UPR (%)** | | | **Gene ID** | **500 bp UPR (%)** | | | **Gene ID** | **GBR (%)** | | |
| --- | --- | --- | --- | --- | --- | --- | --- | --- | --- | --- | --- |
|  | **WT** | ***rdd*** | **ΔML** |  | **WT** | ***rdd*** | **ΔML** |  | **WT** | ***rdd*** | **ΔML** |
| *AT5G47260* | 1.08 | 63.27 | 62.19 | *AT4G11170* | 1.67 | 60.61 | 58.94 | *AT5G45230* | 18.42 | 41.64 | 23.22 |
| *AT4G11170* | 1.69 | 61.4 | 59.71 | *AT5G47260* | 0.26 | 33.75 | 33.49 | *AT4G19530* | 11.52 | 34.08 | 22.56 |
| *AT5G45240* | 0 | 42.47 | 42.47 | *AT5G47250* | 0.24 | 32.03 | 31.79 | *AT1G59780* | 25.63 | 47.26 | 21.63 |
| *AT5G35450* | 66.25 | 95 | 28.75 | *AT1G59780* | 0.07 | 31.04 | 30.97 | *AT4G11170* | 23.63 | 43.85 | 20.22 |
| *AT3G46710* | 62.22 | 89.94 | 27.72 | *AT4G27190* | 0.77 | 31.42 | 30.65 | *AT1G63870* | 31.89 | 51.85 | 19.96 |
| *AT5G36930* | 68.13 | 95.51 | 27.38 | *AT5G40060* | 33.62 | 62.31 | 28.69 | *AT4G16920* | 56.22 | 75.62 | 19.4 |
| *AT2G14080* | 60.14 | 75.52 | 15.38 | *AT5G45240* | 0.86 | 27.02 | 26.16 | *AT4G16960* | 09.21 | 28.3 | 19.09 |
| *AT5G44870* | 62.85 | 76.16 | 13.31 | *AT5G35450* | 72.9 | 96.89 | 23.99 | *AT4G16890* | 20.53 | 39.45 | 18.92 |
| *AT5G38350* | 22.22 | 0.78 | -21.44 | *AT1G31540* | 0.19 | 24.14 | 23.95 | *AT2G17060* | 0.88 | 19.33 | 18.45 |
|  |  |  |  | *AT3G07040* | 37.82 | 61.65 | 23.83 | *AT4G16950* | 22.51 | 40.95 | 18.44 |
|  |  |  |  | *AT2G17060* | 4.13 | 24.59 | 20.46 | *AT5G45260* | 20.27 | 38.53 | 18.26 |
|  |  |  |  | *AT4G16960* | 1.14 | 20.21 | 19.07 | *AT4G09430* | 16.68 | 32.8 | 16.12 |
|  |  |  |  | *AT3G46710* | 72.44 | 91.21 | 18.77 | *AT2G14080* | 3.39 | 19.27 | 15.88 |
|  |  |  |  | *AT1G12280* | 50.5 | 67.45 | 16.95 | *AT4G08450* | 28.4 | 44.06 | 15.66 |
|  |  |  |  | *AT2G14080* | 66.33 | 82.5 | 16.17 | *AT1G56510* | 13.97 | 28.35 | 14.38 |
|  |  |  |  | *AT5G36930* | 78.81 | 94.95 | 16.14 | *AT4G16900* | 37.21 | 50.82 | 13.61 |
|  |  |  |  | *AT1G61180* | 0.18 | 15.24 | 15.06 | *AT5G41750* | 5.26 | 18.69 | 13.43 |
|  |  |  |  | *AT5G51630* | 0.45 | 14.12 | 13.67 | *AT4G16860* | 66.81 | 79.66 | 12.85 |
|  |  |  |  | *AT3G46530* | 0.11 | 13.77 | 13.66 | *AT1G63750* | 6.8 | 18.24 | 11.44 |
|  |  |  |  | *AT5G17890* | 67.41 | 79.86 | 12.45 | *AT5G36930* | 35.78 | 47.16 | 11.38 |
|  |  |  |  | *AT4G33300* | 0.36 | 12.39 | 12.03 | *AT4G33300* | 41.16 | 52.26 | 11.1 |
|  |  |  |  | *AT5G46490* | 9.52 | 19.75 | 10.23 | *AT1G58602* | 74.16 | 84.97 | 10.81 |

**(Continued)**

| **Gene ID** | **200 bp UPR (%)** | | | **Gene ID** | **500 bp UPR (%)** | | | **Gene ID** | **GBR (%)** | | |
| --- | --- | --- | --- | --- | --- | --- | --- | --- | --- | --- | --- |
|  | **WT** | ***rdd*** | **ΔML** |  | **WT** | ***rdd*** | **ΔML** |  | **WT** | ***rdd*** | **ΔML** |
|  |  |  |  | *AT2G17050* | 57.65 | 0.62 | -57.03 | *AT3G14470* | 30.56 | 41 | 10.44 |
|  |  |  |  |  |  |  |  | *AT5G46470* | 41.04 | 50.88 | 9.84 |
|  |  |  |  |  |  |  |  | *AT5G45050* | 26.48 | 35.8 | 9.32 |
|  |  |  |  |  |  |  |  | *AT3G44670* | 0.49 | 9.54 | 9.05 |
|  |  |  |  |  |  |  |  | *AT2G16870* | 19.92 | 28.83 | 8.91 |
|  |  |  |  |  |  |  |  | *AT1G53350* | 17.56 | 25.97 | 8.41 |
|  |  |  |  |  |  |  |  | *AT3G50950* | 41.27 | 48.44 | 7.17 |
|  |  |  |  |  |  |  |  | *AT5G44870* | 2.67 | 9.82 | 7.15 |
|  |  |  |  |  |  |  |  | *AT4G19520* | 2.94 | 10.04 | 7.1 |
|  |  |  |  |  |  |  |  | *AT3G46730* | 7.05 | 14.1 | 7.05 |
|  |  |  |  |  |  |  |  | *AT4G14370* | 8.03 | 14.93 | 6.9 |
|  |  |  |  |  |  |  |  | *AT5G43740* | 4.13 | 10.75 | 6.62 |
|  |  |  |  |  |  |  |  | *AT1G12290* | 44.4 | 50.87 | 6.47 |
|  |  |  |  |  |  |  |  | *AT1G63730* | 23.69 | 30.13 | 6.44 |
|  |  |  |  |  |  |  |  | *AT5G58120* | 4.1 | 10.42 | 6.32 |
|  |  |  |  |  |  |  |  | *AT5G41740* | 1.52 | 7.26 | 5.74 |
|  |  |  |  |  |  |  |  | *AT2G17050* | 2 | 7.5 | 5.5 |
|  |  |  |  |  |  |  |  | *AT1G17600* | 5.48 | 10.79 | 5.31 |
|  |  |  |  |  |  |  |  | *AT5G05400* | 16 | 21.28 | 5.28 |
|  |  |  |  |  |  |  |  | *AT3G04220* | 6.13 | 1.13 | -5 |
|  |  |  |  |  |  |  |  | *AT4G19050* | 24.71 | 17.41 | -7.3 |

UPR: upstream region; GBR: gene body region; listed are the *NLR* genes whose methylation difference between the WT and mutants is > 10% within the UPR or > 5% within the GBR.
