# Supplementary material for: Impact of DNA Demethylases on the DNA Methylation and Transcription of Arabidopsis NLR Genes
Source: Front Genet. 2020 May 26;11:460. doi: 10.3389/fgene.2020.00460 (PMC7264425; doi:10.3389/fgene.2020.00460)
Supplement: Supplementary file 7 [file Table_7.DOCX]

**TABLE S7 |** Transcriptional activity of some Arabidopsis *NLR* genes from wild-type and *ros1* mutants. The abundance of the *NLR* transcripts was estimated and normalized with the value of FPKM (the expected fragments per kilobase of a transcript per million fragments sequenced), by Cufflinks software (v.2.2.1) (Trapnell et al. 2012).

| **Gene ID** | **FPKM** | | |
| --- | --- | --- | --- |
|  | **WT** | ***ros1*** | **Ratio** |
| *AT4G19520* | 2.7169 | 5.3407 | 1.97 |
| *AT5G44510* | 3.0324 | 3.9616 | 1.31 |
| *AT1G12220* | 1.4666 | 1.8937 | 1.29 |
| *AT5G40060* | 1.1156 | 1.4322 | 1.28 |
| *AT1G12280* | 1.2334 | 1.534 | 1.24 |
| *AT1G31540* | 3.1247 | 3.8725 | 1.24 |
| *AT4G12020* | 1.7679 | 2.1521 | 1.22 |
| *AT3G07040* | 1.2156 | 1.4709 | 1.21 |
| *AT1G61180* | 4.0778 | 4.8885 | 1.2 |
| *AT5G43730* | 0.8887 | 1.0698 | 1.2 |
| *AT5G46490* | 1.6649 | 2.0009 | 1.2 |
| *AT1G57630* | 2.2357 | 2.6454 | 1.18 |
| *AT1G56520* | 1.6275 | 1.904 | 1.17 |
| *AT5G11250* | 1.9075 | 2.228 | 1.17 |
| *AT5G66910* | 2.779 | 3.2438 | 1.17 |
| *AT5G46270* | 2.9179 | 3.3941 | 1.16 |
| *AT1G63860* | 3.1308 | 3.5976 | 1.15 |
| *AT4G16860* | 6.5887 | 7.5971 | 1.15 |
| *AT5G46470* | 4.9694 | 5.7002 | 1.15 |
| *AT1G63740* | 2.0328 | 2.3171 | 1.14 |
| *AT1G72840* | 1.0367 | 1.1852 | 1.14 |
| *AT3G44480* | 6.175 | 7.0293 | 1.14 |
| *AT5G40910* | 8.7511 | 9.939 | 1.14 |
| *AT4G16900* | 4.4552 | 5.0381 | 1.13 |
| *AT4G19510* | 6.3863 | 7.1964 | 1.13 |
| *AT5G45250* | 3.7868 | 4.2538 | 1.12 |
| *AT5G46510* | 2.8329 | 3.1848 | 1.12 |
| *AT3G44630* | 3.0538 | 3.381 | 1.11 |
| *AT5G35450* | 1.6712 | 1.8628 | 1.11 |
| *AT5G38850* | 2.4274 | 2.6898 | 1.11 |
| *AT5G45260* | 2.7073 | 3.0102 | 1.11 |
| *AT1G69550* | 2.2022 | 2.4131 | 1.1 |
| *AT3G14470* | 1.727 | 1.8932 | 1.1 |
| *AT4G16950* | 8.1413 | 8.9387 | 1.1 |
| *AT4G19500* | 5.6863 | 6.2749 | 1.1 |
| *AT5G22690* | 2.0677 | 2.2764 | 1.1 |
| *AT5G44870* | 3.4555 | 3.8053 | 1.1 |
| *AT5G66900* | 7.4814 | 8.2184 | 1.1 |

**(Continued)**

| **Gene ID** | **FPKM** | | |
| --- | --- | --- | --- |
|  | **WT** | ***ros1*** | **Ratio** |
| *AT1G58602* | 24.4325 | 22.0922 | 0.9 |
| *AT1G10920* | 1.5106 | 1.3495 | 0.89 |
| *AT1G63750* | 3.0222 | 2.7006 | 0.89 |
| *AT1G62630* | 1.5488 | 1.2954 | 0.84 |
| *AT1G59620* | 2.2221 | 1.6281 | 0.73 |

Listed are the *NLR* genes whose expressions are no less than one FPKM in WT or mutants and their ratios are ≥ 1.1 or ≤ 0.9.
