# Supplementary material for: Impact of DNA Demethylases on the DNA Methylation and Transcription of Arabidopsis NLR Genes
Source: Front Genet. 2020 May 26;11:460. doi: 10.3389/fgene.2020.00460 (PMC7264425; doi:10.3389/fgene.2020.00460)
Supplement: Supplementary file 8 [file Table_8.DOCX]

**TABLE S8 |** Transcriptional activity of some Arabidopsis *NLR* genes from wild-type and *rdd* mutants. The abundance of the *NLR* transcripts was calculated as shown in Table S7. FPKM, the expected fragments per kilobase of a transcript per million fragments sequenced.

| **Gene ID** | **FPKM** | | |
| --- | --- | --- | --- |
|  | **WT** | ***rdd*** | **Ratio** |
| *AT1G12280* | 1.5344 | 1.9792 | 1.29 |
| *AT1G61180* | 3.415 | 4.0868 | 1.2 |
| *AT4G19520* | 4.3572 | 4.955 | 1.14 |
| *AT5G43470* | 13.9975 | 12.6559 | 0.9 |
| *AT5G46450* | 6.6317 | 5.96 | 0.9 |
| *AT1G12220* | 2.4659 | 2.2 | 0.89 |
| *AT1G31540* | 4.1234 | 3.6608 | 0.89 |
| *AT1G59620* | 2.0904 | 1.8529 | 0.89 |
| *AT2G14080* | 5.4135 | 4.7788 | 0.88 |
| *AT4G19500* | 7.2518 | 6.2923 | 0.87 |
| *AT5G44510* | 3.5374 | 3.0883 | 0.87 |
| *AT5G66900* | 7.9274 | 6.9121 | 0.87 |
| *AT4G19510* | 6.4543 | 5.5306 | 0.86 |
| *AT5G22690* | 1.7105 | 1.4709 | 0.86 |
| *AT1G56520* | 2.3056 | 1.9365 | 0.84 |
| *AT4G12020* | 2.5068 | 2.099 | 0.84 |
| *AT5G40910* | 9.7106 | 8.1567 | 0.84 |
| *AT5G44870* | 4.2252 | 3.4913 | 0.83 |
| *AT1G61190* | 9.0666 | 7.3363 | 0.81 |
| *AT5G48620* | 16.8967 | 13.7631 | 0.81 |
| *AT3G07040* | 1.6013 | 1.2867 | 0.8 |
| *AT3G44630* | 3.9979 | 3.1882 | 0.8 |
| *AT4G16890* | 17.1814 | 13.7852 | 0.8 |
| *AT5G04720* | 23.8696 | 19.2008 | 0.8 |
| *AT5G45250* | 5.5244 | 4.4128 | 0.8 |
| *AT5G45260* | 3.377 | 2.7055 | 0.8 |
| *AT5G38850* | 3.0749 | 2.4016 | 0.78 |
| *AT4G12010* | 2.026 | 1.562 | 0.77 |
| *AT1G56510* | 4.1966 | 3.1549 | 0.75 |
| *AT1G58602* | 28.4806 | 21.418 | 0.75 |
| *AT1G63740* | 2.405 | 1.8096 | 0.75 |
| *AT5G17890* | 6.3993 | 4.7974 | 0.75 |
| *AT1G12290* | 1.3129 | 0.9677 | 0.74 |
| *AT1G53350* | 3.596 | 2.6465 | 0.74 |
| *AT3G14470* | 3.1306 | 2.2941 | 0.73 |
| *AT4G33300* | 26.1749 | 18.9786 | 0.73 |
| *AT5G45050* | 5.202 | 3.8071 | 0.73 |

**(Continued)**

| **Gene ID** | **FPKM** | | |
| --- | --- | --- | --- |
|  | **WT** | ***rdd*** | **Ratio** |
| *AT1G72840* | 1.1998 | 0.8666 | 0.72 |
| *AT5G46270* | 3.7689 | 2.7076 | 0.72 |
| *AT5G66910* | 4.2213 | 2.9386 | 0.7 |
| *AT1G63880* | 9.6879 | 6.4451 | 0.67 |
| *AT3G44480* | 8.2254 | 5.4837 | 0.67 |
| *AT3G50950* | 32.8212 | 21.8134 | 0.66 |
| *AT4G36150* | 1.4993 | 0.9885 | 0.66 |
| *AT5G46490* | 2.4207 | 1.6085 | 0.66 |
| *AT3G44670* | 9.1992 | 5.9677 | 0.65 |
| *AT5G45060* | 3.3769 | 2.1826 | 0.65 |
| *AT1G62630* | 2.4675 | 1.5432 | 0.63 |
| *AT1G33560* | 5.5726 | 3.4799 | 0.62 |
| *AT5G41740* | 2.0871 | 1.2563 | 0.6 |
| *AT1G69550* | 3.0384 | 1.7844 | 0.59 |
| *AT5G17880* | 5.0239 | 2.9498 | 0.59 |
| *AT5G36930* | 2.7069 | 1.5879 | 0.59 |
| *AT1G63350* | 1.8209 | 1.0346 | 0.57 |
| *AT1G15890* | 1.0931 | 0.5982 | 0.55 |
| *AT1G63860* | 3.4065 | 1.8157 | 0.53 |
| *AT1G57630* | 17.1693 | 8.7883 | 0.51 |
| *AT5G45510* | 46.1119 | 20.5564 | 0.45 |
| *AT5G46520* | 1.6555 | 0.7525 | 0.45 |
| *AT3G44400* | 1.4254 | 0.604 | 0.42 |
| *AT5G41750* | 2.6265 | 0.951 | 0.36 |
| *AT5G38340* | 1.1179 | 0.3632 | 0.32 |
| *AT5G58120* | 1.7956 | 0.5701 | 0.32 |
| *AT4G11170* | 1.1865 | 0.2373 | 0.2 |

Listed are the *NLR* genes whose expressions are no less than one FPKM in WT or mutants and their ratios are ≥1.1 or ≤ 0.9.
