# Supplementary material for: Impact of DNA Demethylases on the DNA Methylation and Transcription of Arabidopsis NLR Genes
Source: Front Genet. 2020 May 26;11:460. doi: 10.3389/fgene.2020.00460 (PMC7264425; doi:10.3389/fgene.2020.00460)
Supplement: Supplementary file 9 [file Table_9.DOCX]

**TABLE S9 |** *NLR* genes with common expression patterns in different demethylase mutants. The data of these several highlighted genes are from Tables S7 and S8, and the ratio values were calculated with their FPKM values in different genotype.

| **Gene ID** | **Ratio (Transcriptional activity)** | |
| --- | --- | --- |
|  | ***ros1***/WT | ***rdd/*WT** |
| *AT1G12280* | 1.24 | 1.29 |
| *AT1G61180* | 1.2 | 1.2 |
| *AT4G19520* | 1.97 | 1.14 |
| *AT1G58602* | 0.9 | 0.75 |
| *AT1G59620* | 0.73 | 0.89 |
| *AT1G62630* | 0.84 | 0.63 |
